# Body temperature correlates with mortality in COVID-19 patients

**DOI:** 10.1186/s13054-020-03045-8

**Published:** 2020-06-05

**Authors:** Serena Tharakan, Koichi Nomoto, Satoshi Miyashita, Kiyotake Ishikawa

**Affiliations:** Cardiovascular Research Center, Icahn School of Medicine at Mount Sinai, One Gustave L. Levy Place, Box 1030, New York, NY 10029-6574 USA

Systemic inflammation that accompanies acute respiratory distress syndrome in COVID-19 [1] is associated with a high mortality rate, as high as 32.5% [2]. Treatment options for severe cases remain limited [3]. The high mortality rate, lack of effective therapies, and extremely high volume of cases have led to a clear need for reliable prognostic markers to indicate which patients are at the highest risk of death and thus require closer monitoring.

One factor that is common to the majority of hospitalized COVID-19 patients is fever. The degree of temperature elevation might reflect the severity of inflammation. However, there are currently no published studies that have looked at body temperature (BT) as a potential prognostic marker. We sought to analyze how BT monitoring might inform mortality rate estimates in COVID-19-positive patients.

We analyzed BT data in the de-identified database of COVID-19-suspected patients in Mount Sinai and its affiliated hospitals in the New York area as of May 3, 2020. A total of 9417 patients tested positive for the SARS-CoV-2 virus by RT-PCR detection. After excluding patients with missing temperature data (*n* = 1802), 7614 patients were included in the analysis (Table 1). Fifty percent had a BT > 37 °C on the initial presentation and 78.5% of patients developed BT > 37 °C during the course of the disease. The overall mortality was 16.9% with a median of 7 days to death from the initial presentation. As shown in Fig. 1a, higher BT at the initial presentation did not show a significant association to mortality. Importantly, patients presenting with BT ≤ 36 °C had the highest mortality (26.5%, *P* = 0.003 relative to 36 °C < BT ≤ 37 °C), and this became even higher when the analysis was restricted to those with BT ≤ 35.5 °C (44%), indicating low body temperature at the initial presentation is a marker of poor prognosis. Meanwhile, maximum BT during COVID-19 infection was significantly correlated with mortality rate (Fig. 1b). There was a significant increase in mortality for every 0.5 °C increase in BT, and the mortality was as high as 42% in those with maximum BT > 40.0 °C*.*

**Table 1:** Characteristics of patients at first encounter

	Total population (*n* = 7614)	Died (*n* = 1286)	Alive (*n* = 6328)
Age	59.4 ± 18.4	73.8 ± 12.5	56.5 ± 18.1*
Sex (% male)	54.2	59.5	53*
Body mass index	28.8 ± 7.4	29.0 ± 7.8	28.8 ± 7.3
Discharged alive (%)	/	/	83
Temperature (°C)	37.0 (36.7, 37.7)	37.0 (36.6, 37.7)	37.0 (36.7, 37.7)
Systolic blood pressure (mmHg)	131 ± 23.2	128.7 ± 27.8	131.6 ± 22.0*
Diastolic blood pressure (mmHg)	75.3 ± 13.8	71.7 ± 15.9	76.1 ± 13.2*
Heart rate (BPM)	95.2 ± 19.5	96.8 ± 21.9	94.9 ± 18.9*
Oxygen saturation (%)	96 (94, 98)	94 (88, 97)	97 (94, 99)*

Demographic and vitals for 7614 patients who tested positive for the SARS-CoV-2 virus by RT-PCR detection. Data are reported in mean ± SD, with the exception of oxygen saturation (median, interquartile range (IQR))

*P < 0.01 for those who died vs alive

**Fig. 1:**
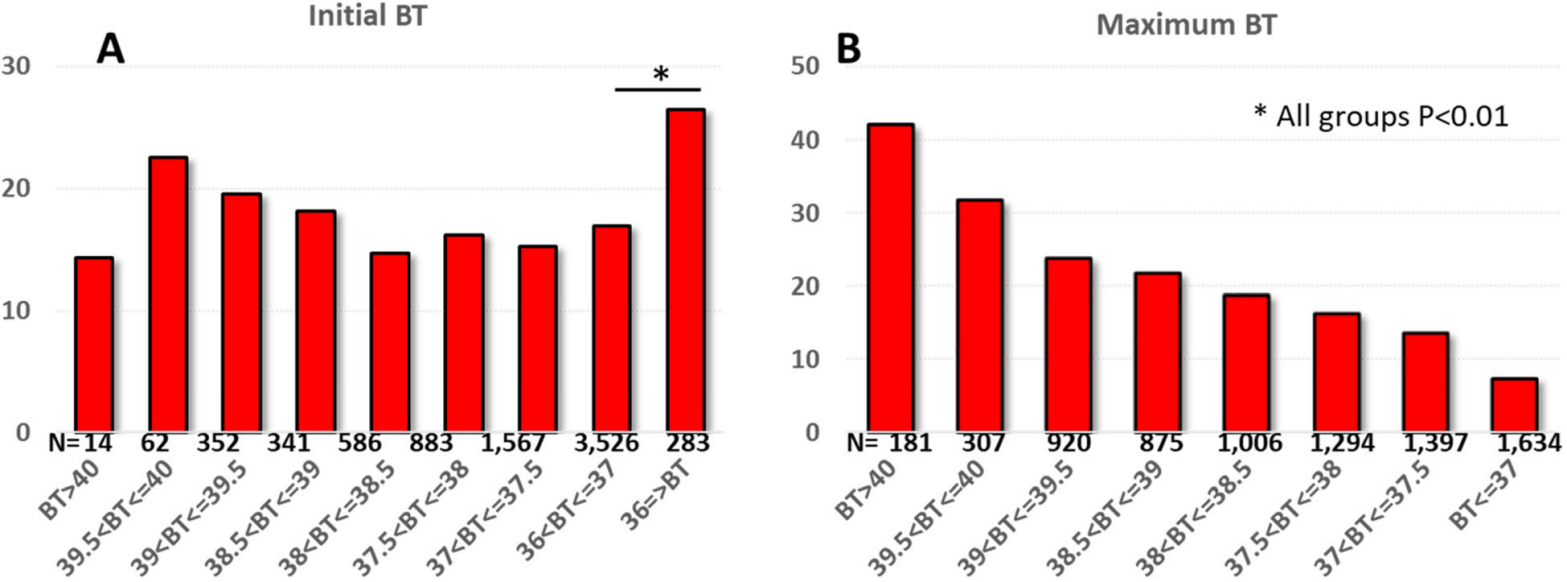
Body temperature and mortality in COVID-19 patients. **a** High body temperature at the initial presentation did not correlate significantly with mortality. Patients with BT ≤ 36 °C had significantly higher mortality compared to normothermia patients. **b** There was a significant increase in mortality for every 0.5 °C increase in maximum BT during the course of COVID-19 (Holms test)

Our results indicate that only half of the patients with positive SARS-CoV-2 virus present with BT > 37 °C at the initial presentation. However, temperature elevation is common, and high maximum temperature during the course of SARS-CoV-2 infection was a significant harbinger of poor outcomes. In fact, one in three patients reaching a maximum BT above 39.5 °C died. This was approximately a 5-fold increase in mortality rate as compared to patients whose temperature never broke 37 °C. In contrast, almost half of the patients initially presenting with low BT (< 35.5 °C) died. Our results, therefore, suggest that poor BT control during the COVID 19 disease course is a marker of poor prognosis and BT can be used as an easily obtained prognostic indicator.

It remains unknown if controlling the high temperature in severely ill COVID-19 patients would alleviate inflammatory response and improve their outcome. Future studies are necessary to address this question. We acknowledge the limitations of our study including unknown methods of temperature measurement, lack of follow-up of temperature in patients without hospital admission, and that the data is not adjusted for potential confounding factors. Nevertheless, a clear trend in increased mortality among the patients with poor temperature control highlights the usefulness of this non-invasively and easily obtained parameter for evaluating patients’ prognoses.

## Data Availability

The datasets generated and/or analyzed during the current study are not publicly available due to institutional policy but are available from the corresponding author on reasonable request.

## Abbreviation

BT: Body temperature
